# Preventive procedure for stenosis after esophagojejunostomy using a circular stapler and transorally inserted anvil (OrVil™) following laparoscopic proximal gastrectomy and total gastrectomy involving reduction of anastomotic tension

**DOI:** 10.1186/s12893-021-01054-0

**Published:** 2021-01-21

**Authors:** Eiji Nomura, Hajime Kayano, Takatoshi Seki, Rin Abe, Hisamichi Yoshii, Shuji Uda, Akihito Kazuno, Hideki Izumi, Soichiro Yamamoto, Masaya Mukai, Hiroyasu Makuuchi

**Affiliations:** Department of Gastroenterological and General Surgery, Tokai University Hachioji Hospital, 1838 Ishikawa-machi, Hachioji, Tokyo, 192-0032 Japan

**Keywords:** Gastric cancer, Laparoscopic proximal gastrectomy, Laparoscopic total gastrectomy, Anastomotic stenosis, Esophagojejunostomy, OrVil™

## Abstract

**Background:**

Recently, due to increasing reports of stenosis after esophagojejunostomy created using circular staplers and a transorally inserted anvil (OrVil™) following laparoscopic proximal gastrectomy (LPG) and total gastrectomy (LTG), linear staplers are being used instead. We investigated our preventive procedure for esophagojejunostomy stenosis following use of circular staplers.

**Methods:**

Since the anastomotic stenosis is considered to be mainly caused by tension in the esophageal and jejunal stumps at the anastomotic site, we have been performing procedures to relieve this tension, by cutting off the rubber band and pushing the shaft of the circular stapler toward the esophageal side, since July 2015. We retrospectively compared the incidence of anastomotic stenosis in cases of LPG and LTG performed before July 2015 (early phase, 30 cases) versus those performed after this period (later phase, 22 cases).

**Results:**

Comparison of the incidence of anastomotic stenosis according to the type of surgery, LPG or LTG, and between the two time periods versus all cases, indicated a significantly lower incidence in the later phase than in the early phase (4.5 vs. 26.7%, *p* < 0.05), especially for LPG (0 vs. 38.5%, *p* < 0.05).

**Conclusions:**

It is possible to use a circular stapler during laparoscopic esophagojejunostomy, as with open surgery, if steps to reduce tension on the anastomotic site are undertaken. These procedures will contribute to the spread of safe and simple laparoscopic anastomotic techniques.

## Background

Although the incidence of gastric cancer is expected to decrease in the future due to the decrease in *H. pylori* infection, the number of gastric cancer patients has been increasing or leveling in Japan due to the aging of patients [1]. Furthermore, an increase in cancers at the upper third of the stomach and the esophagogastric junction (EGJ) has been observed [2, 3], and the incidence of performance of total and/or proximal gastrectomy, which is more difficult than distal gastrectomy, is increasing. With a focus on minimally invasive surgery, laparoscopic gastrectomy has become common for early gastric cancer [4, 5]. Hence, there is demand for a technique that can be safely performed even with more difficult operations and in high-risk patients.

For early gastric cancer limited to the upper third of the stomach, we typically perform laparoscopic proximal gastrectomy (LPG), with reconstruction by the double tract (DT) method or jejunal interposition (JIP) method [5]. For extensive early gastric cancer not localized to the upper third of the stomach or multiple early gastric cancers, laparoscopic total gastrectomy (LTG) with Roux en Y reconstruction is performed [6]. Both of these surgical procedures involve the important step of esophagojejunostomy, which has the potential to cause problems related to the anastomotic site, which have a great impact on postoperative quality of life (QOL) since they can delay oral intake and prolong the duration of hospital stay [7]. Although we perform esophagojejunostomy using a circular stapler (CS) and a transorally inserted anvil (OrVil™, Covidien, Mansfield, MA, USA), many surgeons use a linear stapler and perform reconstruction using functional end to end anastomosis [8, 9] or an overlap method [10, 11] because of the high incidence of occurrence of anastomotic stenosis with laparoscopic surgery [12–14]. On the other hand, esophagojejunostomy using a CS is still commonly used in open total gastrectomy [15, 16]. In this study, we aimed to resolve the uncertainties related to laparoscopic use of a CS.

In terms of the difference between open surgery and laparoscopic surgery, the former provides an adequate field of view, while the latter requires certain special measures to obtain an adequate surgical field. Further, a single stapling technique (SST) using CS is used in open surgery, while the latter uses a hemi-double stapling technique (HDST) with an OrVil™ anvil delivery system. We investigated whether the occurrence of anastomotic stenosis is reduced by minimizing these differences and performing a near-open procedure that reduces anastomotic tension.

## Methods

### Study design and patient selection

This retrospective cohort study was conducted at Tokai University Hachioji Hospital after being approved by the Institutional Review Board for Clinical Research at Tokai University. In this study, we retrospectively evaluated 22 patients who underwent LPG (DT: 16 patients, JIP: 6 patients) and 30 patients who underwent LTG from April 2013 to March 2019. All of these cases were followed up at our outpatient clinic, and upper gastrointestinal endoscopy (UGE) was performed within one year after surgery. If the patient complained of dysphagia, UGE was performed as quickly as possible using a 9.9-mm-diameter endoscope (GIF-Q260J; OLYMPUS Co., Ltd., Tokyo, Japan). In this study, if passage of the GIF-Q260J endoscope was unsuccessful at the first attempt, the esophagojejunostomy orifice was deemed to have developed anastomotic stenosis. In such cases, endoscopic balloon dilatation (EBD) was performed. Briefly, a balloon catheter (CRE wire-guided balloon dilation catheter; Boston Scientific, Natick, MA, USA) was inserted through the endoscope, and the 15/18-mm diameter balloon was gradually filled with water and maintained at an adequate pressure for 1–2 min. After the dilatation, the endoscope was confirmed to easily pass through the anastomotic site without force. If restenosis was recognized, balloon dilatation was repeated until the stenosis was adequately treated.

Since we opined that the anastomotic stricture is caused by tension on the esophageal and jejunal walls, in July 2015 we decided to perform an additional intraoperative procedure to release this tension. The period before we started performing our tension-relieving procedure was considered as the early phase, and the subsequent period was regarded as the later phase. In addition, as described below, since JIP only crimps the jejunum on the caudal side of the gastrojejunostomy during DT, with the procedure for esophagojejunostomy being the same, we compared cases of LPG reconstructed using both JIP or DT versus cases of LTG alone. Anastomotic leakage as a complication related to anastomosis and pancreatic fistula as a complication related to lymph node dissection were also investigated.

Although neoadjuvant chemotherapy (NAC) has been recommended for stage IB-III gastric cancer according to European Society for Medical Oncology (ESMO) Clinical Practice Guidelines [17] and National Comprehensive Cancer Network (NCCN) Guidelines [18], we do not administer it in all cases because the latest Japanese Gastric Cancer Treatment Guideline (JGCTG 2018; ver. 5) recommends NAC only for resectable gastric cancer cases with bulky lymph node metastasis. On the other hand, since postoperative adjuvant chemotherapy with oral tegafur / gimeracil / oteracil (S-1) is recommended for stage II and S-1 ± Oxaliplatin for stage III cases, all such patients were treated in accordance with the guidelines after receiving their consent.

Our surgical procedures and the study protocol were approved by the Human Ethics Review Committees of Tokai University School of Medicine (Institutional Review Board number 14R043). Written, informed consent was obtained from each enrolled patient before the surgery, in accordance with the Declaration of Helsinki.

### Laparoscopic procedure

Patients were placed in the reverse Trendelenburg position with their legs apart. The surgeon stood between the patient's legs, the first assistant (standing on the left side) handled the laparoscope with the left hand and assisted the operator with his right hand. The second assistant (standing on the right side) assisted the operator with his right hand. A vertical incision about 2 cm in length was made at the umbilicus for insertion of a 12 mm port, and the laparoscope was inserted into the port after creation of the pneumoperitoneum. Next, a 5 mm port was inserted on the right and a 12 mm port was inserted on the left, at a position about 6–8 cm lateral to the navel. In addition, a 5 mm port was inserted on the right and a 12 mm port on the left slightly above and 12–14 cm lateral to the navel. Nathanson's retractor inserted just caudal to the xiphoid process was used to elevate the round ligament and the lateral segment of the liver. In all our cases, LPG and LTG were performed with D1 + lymph node dissection according to Japanese treatment guidelines [19]. In principle, D2 lymphadenectomy is indicated for cN + or ≥ cT2 tumors and D1 or D1 + lymphadenectomy is recommended for cT1N0 tumors, according to NCCN guidelines [18]. However, D2 lymph node dissection with total gastrectomy requires additional resection of the spleen in cases needing hilar lymph node dissection according to the JGCTG 2010 guidelines (ver. 3) (English edition), although this procedure is no longer recommended except in cases of total gastrectomy for proximal gastric cancer that does not invade the greater curvature, according to the latest JGCTG guidelines (ver. 5). Following this guideline, our strategy for lymph node dissection in LTG cases is equivalent to the current D2 lymph node dissection.

### LPG

#### DT method

**Fig. 1:**
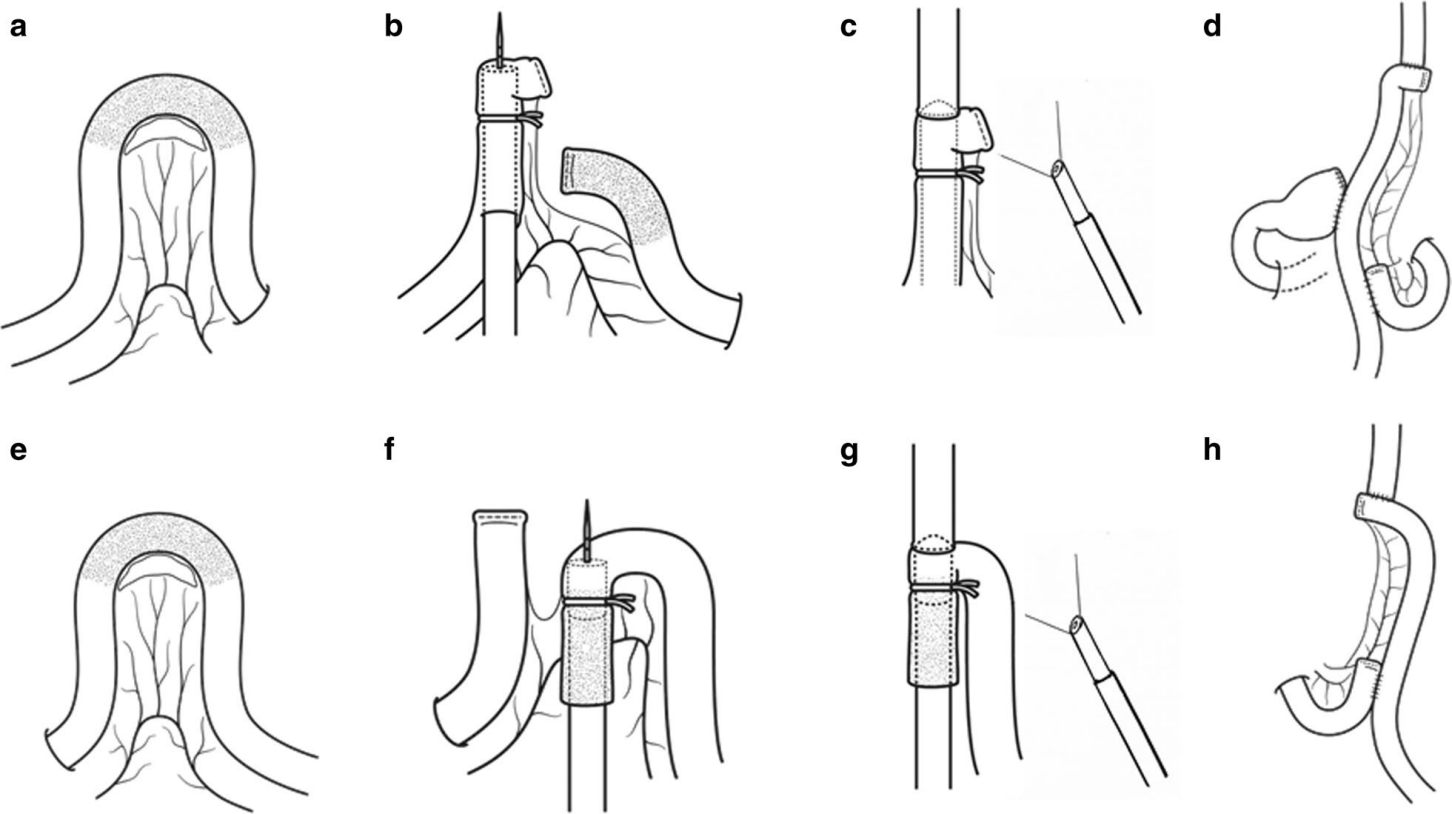
Schematic illustration of the steps of intracorporeal esophagojejunostomy in laparoscopic proximal and total gastrectomy. Laparoscopic proximal gastrectomy: **a** The mesentery supporting the section of the jejunum between 20–30 cm from the ligament of Treitz was dissected along with the jejunum. **b** The head of the shaft of the CS [DST EEA XL™ shaft (Covidien)] was inserted in the cranial direction through the incised jejunum, 10–15 cm from the oral end of the jejunal stump, and the central rod was introduced from just at the caudal side of the jejunal stump. **c** The jejunal stump and the head of the shaft were simultaneously inserted into the abdominal cavity, and anastomosis was performed under a good visual field. **d** Completed reconstruction following laparoscopic proximal gastrectomy. Laparoscopic total gastrectomy: **e** The mesentery supporting the section of the jejunum between 20–30 cm from the ligament of Treitz was dissected along with the jejunum to harvest the jejunum for anastomosis. **f** The head of the shaft of the CS was inserted by incising the oral edge of the caudal part of the jejunum of which the mesentery was dissected, and the center rod of the CS was introduced from the jejunum while maintaining its blood flow. **g** Anastomosis was performed under a good visual field. **h** Completed reconstruction following laparoscopic total gastrectomy

In order to resect half to one-third of the proximal stomach, gastric transection was performed using a linear stapler, with the line of dissection extending orally from the pylorus to 10–12 cm along the lesser curvature and 15–17 cm along the greater curvature. The esophagus was transected slightly obliquely from the right to the left side with a linear stapler. At this time, proximal gastrectomy was completed. The umbilical wound was extended longitudinally to a length of 4 cm, and a wound retractor (Alexis Wound Retractor M, Applied Medical, Rancho Santa Margarita, CA, USA) was inserted. The gastrectomy specimen was pulled out extracorporeally through the minilaparotomy and the proximal margin was evaluated. A surgical glove was attached to the wound protector, and pneumoperitoneum was re-established. Next, the OrVil™ system (DST EEA 25; Covidien) was inserted orally toward the right edge of the esophageal stump along with its anvil. Further, the mesentery supporting a section of the jejunum between 20–30 cm from the ligament of Treitz was dissected along with the corresponding jejunum (Fig. 1a), using a linear stapler for the distal jejunal dissection. The jejunum was then pulled out through the minilaparotomy. Next, the head of the shaft of the CS (DST EEA XL™ shaft (Covidien)) passing through a surgical glove was inserted orally through the incised jejunum that had been pulled out from the abdomen, 10–15 cm from the oral end of the jejunal stump, and the central rod was introduced from just at the caudal side of the jejunal stump (Fig. 1b). At this time, a rubber band was used to secure the jejunum to the head of the shaft, to prevent slippage of the jejunal stump from the shaft. A surgical glove was attached to the wound protector, and pneumoperitoneum was re-established. The jejunal stump and the head of the shaft were simultaneously re-inserted into the abdominal cavity (at this time, these were observed using a laparoscope inserted via the 12 mm port on the left flank), and anastomosis was performed under a good visual field (HDST, Fig. 1c). Further, anastomosis was performed using Albert-Lembert sutures between the insertion hole of the CS and the oral edge of the remnant stomach to complete the jejunogastrostomy under direct vision. The resultant anastomotic diameter was approximately 6 cm. Finally, a side-to-side jejunojejunostomy 20 cm from the caudal end of the jejunogastrostomy was performed extracorporeally (Fig. 1d). Petersen’s defect and the mesenteric gap were closed intracorporeally.

#### JIP method

After the DT reconstruction was completed, the jejunum on the caudal side of the jejunogastrostomy was crimped with a knifeless linear stapler to achieve reconstruction by the JIP method.

### LTG

#### Roux en Y method

After the procedures on the side of the greater curvature along with D1 + lymph node dissection were performed, transection of the duodenum 1 cm away from the pyloric ring was performed using a linear stapler. After performing the procedures on the side of the lesser curvature along with D1 + lymph node dissection, the esophagus was slightly obliquely transected from the right to the left side with a linear stapler. At this time, total gastrectomy was completed. The umbilical wound was extended longitudinally to a length of 4 cm, and a wound retractor was inserted. The specimen was pulled out extracorporeally through the minilaparotomy and the proximal margin was evaluated. A surgical glove was attached to the wound protector, and pneumoperitoneum was re-established. Then, the OrVil™ system (DST EEA 25; Covidien) was inserted orally toward the right edge of the esophageal stump and its anvil was positioned. Further, the mesentery supporting a section of the jejunum between 20 and 30 cm away from the ligament of Treitz was dissected along with the corresponding jejunum (Fig. 1e), and the jejunum was pulled out through the minilaparotomy. Then, the jejunum was dissected with a linear stapler at the oral end of the jejunum of which the mesentery was dissected, to obtain the part of the jejunum that connected with the esophageal stump. The head of the shaft of the CS was inserted by incising the oral edge of the caudal side of the jejunum of which the mesentery was dissected, and the center rod of the CS was introduced from the jejunum while maintaining its blood flow (Fig. 1f). As with LPG, to prevent slippage of the jejunal stump from the shaft, a rubber band was used to secure the sacrificed jejunum to the head of the shaft. Next, a pneumoperitoneum was created using surgical gloves, and the jejunum and the head of the shaft were simultaneously inserted into the abdominal cavity (at this time, these were observed with a laparoscope inserted via the 12 mm port in the left flank of the abdomen). Anastomosis was performed under a good visual field (HDST, Fig. 1g). Then, the shaft of the CS was removed, and the jejunum into which the shaft of the CS had been inserted was transected with a linear stapler to complete the esophagojejunostomy. In addition, a side-to-side jejunojejunostomy with a linear stapler was performed extracorporeally to create a Roux-en-Y limb (Fig. 1h). Petersen’s defect and the mesenteric gap were closed intracorporeally.

#### Technique to avoid anastomotic stenosis

**Fig. 2:**
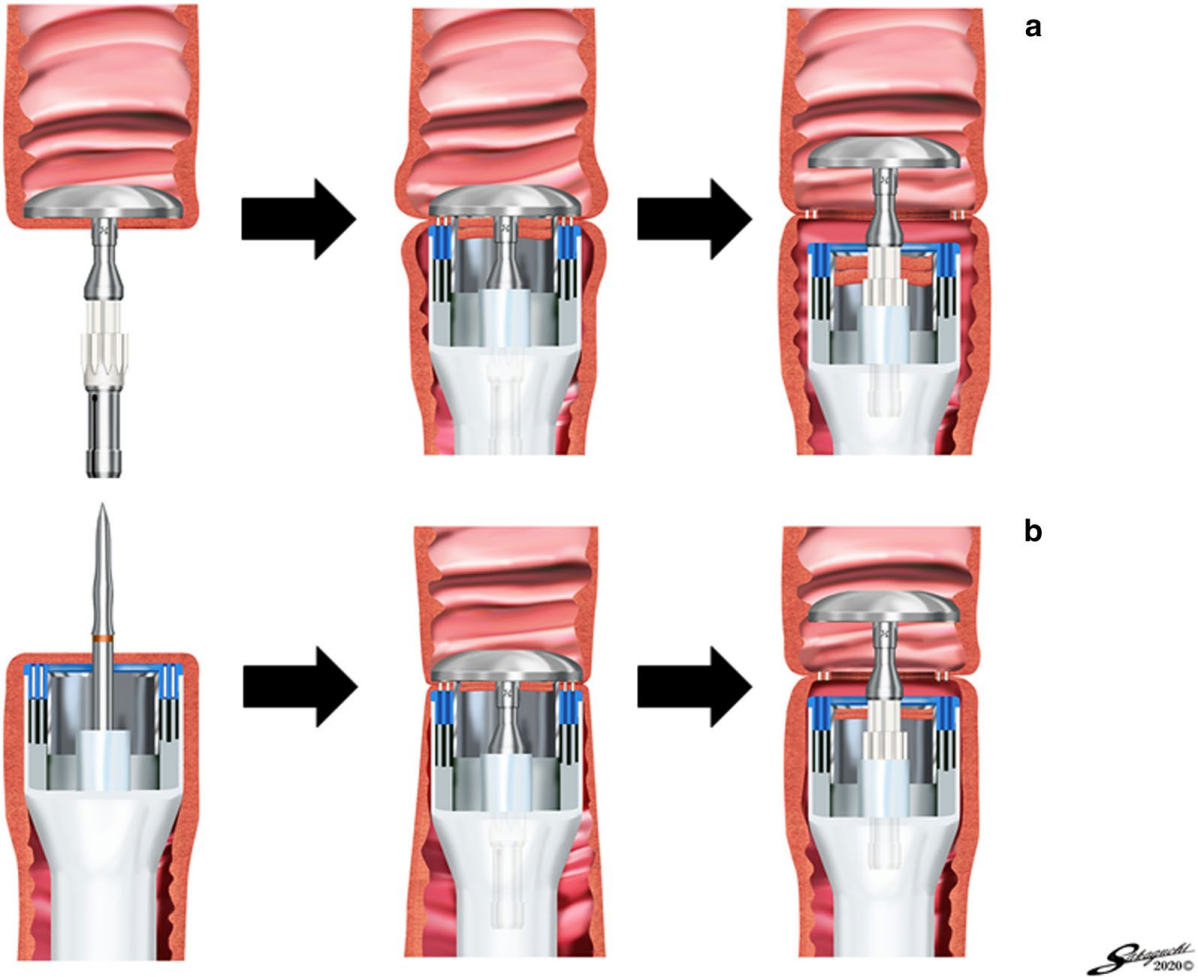
Schematic illustration of our technique to avoid anastomotic stenosis. **a** When the esophagus and jejunum were brought close to each other, the rubber band was cut and the jejunum on the caudal side was pushed toward the cranial side to reduce jejunal tension, and the shaft of the CS was pushed toward the cranial side to reduce esophageal tension. This resulted in a wider anastomotic diameter. **b** If this was not done, the anastomotic diameter was likely to be narrower

**Fig. 3:**
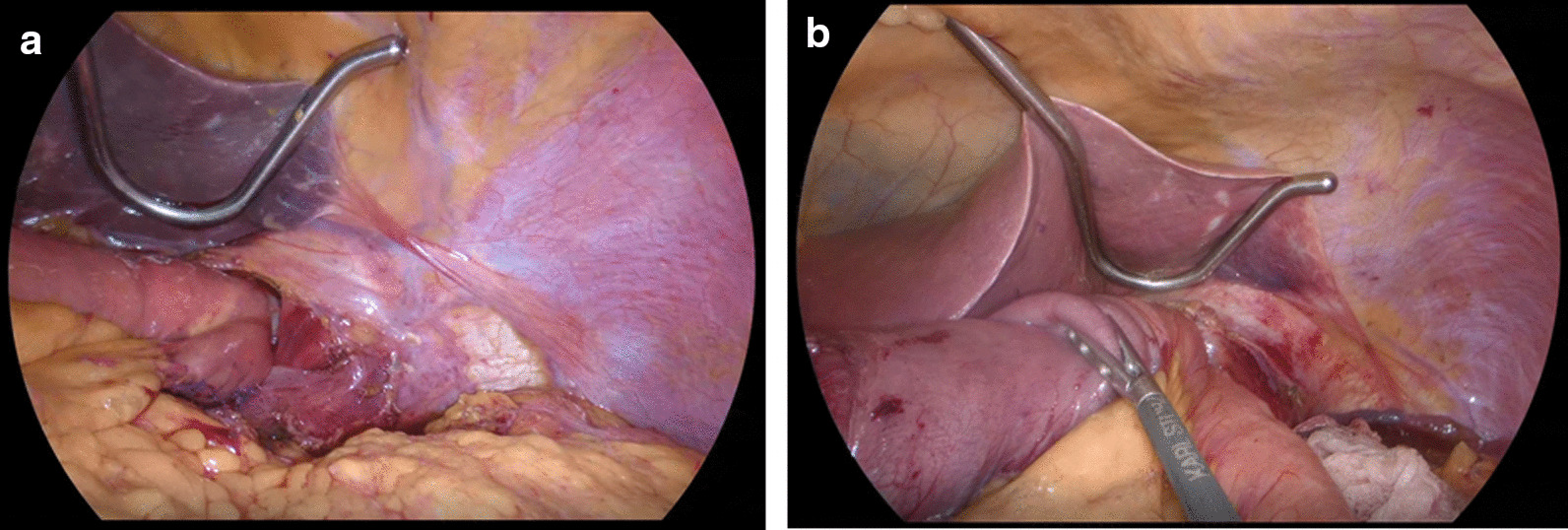
Intracorporeal esophagojejunostomy to avoid anastomotic stenosis. **a** During laparoscopic proximal gastrectomy, the anastomosis is performed after completely connecting the esophageal and jejunal stumps, while reducing the tension on them. **b** The anastomosis is similarly performed after laparoscopic total gastrectomy

Since it is thought that the rubber band used to fix the jejunum to the shaft of the CS (DST EEA XL™ shaft) causes tension on the jejunum, and hauling the shaft of the CS toward the caudal side causes tension on the esophagus, when the esophagus and jejunum were brought close to each other, the rubber band was cut and the jejunum on the caudal side was pushed toward the cranial side to reduce jejunal tension, and the shaft of the CS was pushed toward the cranial side to reduce esophageal tension (Fig. 2a) for both LPG (Fig. 3a) and LTG (Fig. 3b). At this time, it was necessary to be careful that the surrounding tissues were not sandwiched between the esophageal stump and the lifted jejunum. As a result, the anastomotic diameter became wider (Fig. 2a). If this step was not performed, it would result in a narrower anastomotic diameter (Fig. 2b). Since we believed that performing this procedure would eliminate the tension between the esophagus and jejunum and prevent anastomotic stenosis, we decided to perform this procedure with all three reconstructive methods in cases performed after July 2015.

We retrospectively compared the incidence of anastomotic stenosis, anastomotic leakage and pancreatic fistula in cases of LPG (with DT, JIP) and LTG performed before July 2015 (early phase, 30 cases) versus those performed after this period (later phase, 22 cases). Anastomotic stenosis and leakage were assessed using the Clavien-Dindo classification [20]. Furthermore, postoperative pancreatic fistula was assessed according to the International Study Group on Pancreatic Fistula (ISGPF) grading 2016 [21].

### Statistical analysis

Statistical analysis was performed using the *χ*^2^ test, Fisher’s exact test, and Student’s *t*-test. Mann–Whitney’s *U* test was used for multiple comparisons. All statistical analyses were performed using the JMP® software program, ver. 13 (SAS Institute Inc., Cary, NC, USA). A *p* value of less than 0.05 was considered significant.

## Results

The patients’ characteristics and operative findings stratified according to the operative period are shown in Table 1. A total of 52 patients underwent gastrectomy, either LPG or LTG, 30 patients in the early phase and 22 patients in the later phase. In the LTG group, the median follow-up period was 58.2 ± 12.7 (35–73) months in the early phase and 23.6 ± 6.5 (14–34) months in the later phase. In the LPG group, the median follow-up period was 54.8 ± 14.3 (37–74) months in the early phase and 23.8 ± 8.3 (14–35) months in the later phase. There were no clinical dropouts in this patient series, except for two patients, one each in the LTG and LPG groups, who died of diseases unrelated to the gastric cancer. There was no significant difference in background factors between these two groups. Among the complications related to the anastomosis, one patient (4.5%) in the LPG group had Grade IIIb anastomotic leakage (Table 2). Six patients had grade B pancreatic fistula according to the ISGPF grading 2016, constituting 20.0% (6/30) of all patients. Pancreatic fistula might have been common only in the LTG group because of the presence of relatively advanced cancer cases in this group. Grade IIIa anastomotic stenosis was observed in five patients in the LPG group and four patients in the LTG group. When the incidence of anastomotic stenosis was compared in cases of LPG and LTG performed in the early phase (30 cases) versus those performed in the later phase (22 cases), it was significantly lower in the later phase (4.5%) than in the early phase (26.7%) (*p* < 0.05). Subsequently, when LPG and LTG were examined separately, the incidence of anastomotic stenosis in the later phase was significantly reduced in the LPG group (0 vs. 38.5%, *p* < 0.05). In the LTG group, although there was no significant difference between the two phases, anastomotic stenosis in the later phase was found in only one case (7.7% vs. 17.6%). EBD was performed for all these cases as soon as the stenosis was diagnosed endoscopically. EBDs were performed 7.2 ± 6.3 (1–15) times in LPG cases, and 2.3 ± 1.5 (1–4) times in LTG cases. There was one patient in the LPG group with grade IIIb anastomotic leakage that resulted in severe postoperative anastomotic stenosis, in whom EBD was repeated 15 times.

**Table 1:** Characteristics of patients stratified according to operative period

	Early phase (n = 30)	Later phase (n = 22)	*P* value
Age (years)	69.1 ± 9.0	68.3 ± 9.6	N.S
Sex (Male: Female)	24:6	15:7	N.S
Body mass index (kg/m^2^)	22.7 ± 2.9	22.8 ± 3.4	N.S
Operative time (min)	354.9 ± 48.7	362.0 ± 47.5	N.S
Blood loss (mL)	102.8 ± 118.9	104.5 ± 105.6	N.S
LPG, DT	8	8	N.S
LPG, JIP	5	1	
LTG (no. of cases)	17	13	
Time to liquid diet (days)	7.9 ± 7.7	7.2 ± 5.3	N.S
Hospital stay (days)	17.3 ± 12.5	16.7 ± 12.2	N.S
LPG			
Stage IA	8	6	N.S
IB	3	2	
IIA	0	1	
IIB	2	0	
LTG			
Stage IA	11	3	N.S
IB	1	3	
IIA	2	3	
IIB	2	3	
IIIA	1	1	

*LPG* Laparoscopic proximal gastrectomy, *DT* Double tract method, *JIP* Jejunal interposition method, *LTG* Laparoscopic total gastrectomy, *N.S.* not significant

**Table 2:** Incidence rate of postoperative complications according to the operative method and study period

Op. method	Period	Complications		
		Anastomotic stenosis	Anastomotic leakage	Pancreatic fistula
LPG + LTG	Early phase Later phase	8/30 (26.7)^a^ 1/22 (4.5)^b^	1/30 (3.3) 0/22 (0)	4/30 (13.3) 2/22 (9.1)
LPG	Early phase Later phase	5/13 (38.5)^c^ 0/9 (0)^d^	1/13 (7.7) 0/9 (0)	0/13 (0) 0/9 (0)
LTG	Early phase Later phase	3/17 (17.6) 1/13 (7.7)	0/17 (0) 0/13 (0)	4/17 (23.5) 2/13 (15.4)

^a^vs.^b^ and ^c^vs.^d^,* p* < 0.05 (Fisher’s exact test), *LPG* Laparoscopic proximal gastrectomy, *LTG* Laparoscopic total gastrectomy

Anastomotic stenosis and leakage were assessed according to the Clavien-Dindo classification, and postoperative pancreatic fistula was assessed according to the ISGPF grading 2016

## Discussion

With the increase in the number of patients with gastric cancer in the upper third of the stomach and at the EGJ, LPG and LTG have been more frequently performed as minimally invasive surgery for maintenance of postoperative QOL [6, 22, 23]. Furthermore, the procedures allow performance of a safe reconstruction method with less complications. With proximal gastrectomy in particular, the JIP and DT reconstruction methods, in which the jejunum is placed between the lower esophagus and remnant stomach, have been actively performed in order to prevent reflux esophagitis. At some institutes, esophagogastrostomy is performed by the double flap technique to prevent reflux [24, 25], although if the remnant stomach is too small, the JIP method or DT method is used instead of reconstruction. In esophagojejunostomy, anastomosis using the OrVil™ system was previously frequently reported [26, 27]. However, due to the high incidence of anastomotic stenosis that has been reported, a number of institutes have recently been performing the overlap method or functional end-to-end method using a linear stapler. On the other hand, at the time of open surgery, esophagojejunostomy using a CS is commonly and usually used, with subsequent anastomotic stenosis occurring only very rarely [15]. So, why is stenosis of the esophagojejunostomy more of a problem in laparoscopic surgery? Generally, anastomotic stenosis occurs secondary to scar contracture or anastomotic leakage-induced stricture [28]. Stenosis after use of the OrVil™ system is mostly due to scar contracture and usually improves with balloon dilatation. However, the scar contracture usually occurs following specific steps of the anastomotic procedure. The cause of the anastomotic stenosis in esophagojejunostomy using DST or HDST is thought to be tension at the anastomotic site due to traction on the Roux limb [14, 22], or ischemia at the site where the staple lines meet, both of which might lead to fibrosis [14]. However, since we believe that tension on the anastomotic site during anastomotic procedures might be the main cause of fibrosis, as shown in Fig. 2a, we devised procedures to reduce tension at the anastomotic site at the time of anastomosis. This resulted in complete absence of anastomotic stenosis after LPG, with a decrease in the tendency to anastomotic stenosis after LTG, although there was no significant difference as compared to the early period.

Fukagawa et al. [15] described an incidence rate of 4.1% for stenosis of the esophagojejunostomy after open gastrectomy, which was more common in women, with proximal gastrectomy, and with the use of a narrow-sized stapler. In our study, anastomotic stenosis with LTG was observed in two males and two females, although all cases of anastomotic stenosis following LPG occurred in males. Since we used a 25 mm OrVil™ system in all cases, we could not compare the results in terms of the size of the stapler. Comparison of postoperative complications showed that the incidence of pancreatic fistulas was higher in LTG cases, probably because cancer stage was slightly higher in patients who underwent LTG rather than LPG. On the other hand, the incidence of anastomotic stenosis was 22.7% (5/22) in patients who underwent LPG, and was slightly higher than the incidence of 13.3% (4/30) in LTG patients. Fukagawa et al. [15] reported that the incidence rates of anastomotic stenosis were 7.8% in open proximal gastrectomy, and 3.4% in open total gastrectomy. This suggests that anastomotic stenosis might be observed in both laparoscopic and open proximal gastrectomy. On the other hand, it is worth noting that the procedure for avoiding anastomotic stenosis was particularly effective in LPG cases. However, we cannot make a simple comparison in our study because the insertion site in the jejunum into which the CS was inserted was different between LTG and LPG, although it is possible that anastomotic stenosis was reduced by reducing anastomotic tension in both types of surgeries.

Shim et al. [29] performed and compared four types of intracorporeal esophagojejunostomy: conventional anvil head method (the anvil was retrogradely inserted into the esophageal stump), the OrVil™ system method, a hemi-double stapling technique with anvil head (the anvil head is inserted after making a small hole in the anterior wall of the stomach) and side to side esophagojejunostomy with a linear stapler for esophagojejunostomy after LTG. The former two methods might be preferable when the tumor is close to the EGJ, and the latter two methods might be preferable when the tumor is at least 3 cm caudal to the EGJ. Although this indication might not be currently applicable due to recent advances in surgical procedures at the inferior mediastinum [30], it is certainly possible that the esophagojejunostomy procedure that we usually follow for LPG allows easy anastomosis higher in the mediastinum. However, due to the reported risk of contamination by oral bacteria during insertion of the anvil head, it seems necessary to consider the cleanliness of the oral cavity and perform careful device insertion.

Our study has several limitations. This was a single-institution study that involved only a small number of cases. However, the most important limitation was related to the learning curve [10]. Since the incidence of anastomotic stenosis was divided between early and later periods for comparison, we cannot rule out the fact that improvement in technical skills had significant influence on the results. However, it is worth noting that the incidence of anastomotic stenosis dramatically decreased before and after taking measures to relieve anastomotic tension. Certainly, since there were many cases of anastomotic stenosis in the early phase, although we initially thought of switching from anastomosis with a CS to that with a linear stapler, we no longer felt the need to do so in the later period. Hence, we believe our anastomotic technique allowed esophagojejunostomy, which is the most stressful part of the latter half of the operation, to be easily performed using the CS, as in open gastrectomy. However, further randomized clinical trials comparing groups with and without techniques to reduce tension will be needed to verify the procedures that might be effective in avoiding anastomotic stenosis.

## Conclusions

It is possible to use a CS for esophagojejunostomy during laparoscopic gastrectomy, as in open surgery, if procedures to reduce tension on the anastomotic site are undertaken. These procedures will likely contribute to the spread of safe and simple anastomotic techniques.

## Data Availability

The datasets used and/or analyzed during the current study are available from the corresponding author on reasonable request.

## Abbreviations

LPG: Laparoscopic proximal gastrectomy
DT: Double tract method
JIP: Jejunal interposition method
LTG: Laparoscopic total gastrectomy
QOL: Quality of life
CS: Circular stapler
SST: Single stapling technique
HDST: Hemi-double stapling technique
UGE: Upper gastrointestinal endoscopy
EBD: Endoscopic balloon dilatation
NAC: Neoadjuvant chemotherapy
ESMO: European Society for Medical Oncology
NCCN: National Comprehensive Cancer Network
JGCTG: Japanese gastric cancer treatment guideline
ISGPF: International Study Group on Pancreatic Fistula
